# Serological Investigation and Epidemiological Analysis of Bovine Leptospirosis in Egypt

**DOI:** 10.3390/tropicalmed7090208

**Published:** 2022-08-24

**Authors:** Nermin Awade Ibrahim, Barakat M. Alrashdi, Yasser Fathi Elnaker, Ehab Kotb Elmahallawy, Mohamed A. Alblihed, Mohamed said Daib, Amany M. Abd Elmoety, Eman A. Abo Elfadl, Basma M. Badawy, Elzahara Elbaz

**Affiliations:** 1 Bacteriology, Mycology and Immunology Department, Faculty of Veterinary Medicine, Mansoura University, Mansoura 35516, Egypt; 2 Biology Department, College of Science, Jouf University, Sakaka 72388, Saudi Arabia; 3 Animal Medicine Department (Infectious Diseases), Faculty of Veterinary Medicine, New Valley University, El Kharga 72511, Egypt; 4 Department of Zoonoses, Faculty of Veterinary Medicine, Sohag University, Sohag 82524, Egypt; 5 Department of Microbiology, College of Medicine, Taif University, P.O. Box. 11099, Taif 21944, Saudi Arabia; 6 Animal Hygiene and Zoonosis Department, Faculty of Veterinary Medicine, New Valley University, El Kharga 72511, Egypt; 7 Department of Animal Husbandry and Development of Animal Wealth, Faculty of Veterinary Medicine, Mansoura University, Mansoura 35516, Egypt; 8 Department of Hygiene and Zoonoses, Faculty of Veterinary Medicine, Mansoura University, Mansoura 35516, Egypt; 9 Internal Medicine and Infectious Diseases and Fish Diseases Department, Faculty of Veterinary Medicine, Mansoura University, Mansoura 35516, Egypt

**Keywords:** leptospirosis, bovine, epidemiology, serology, Egypt

## Abstract

Bovine leptospirosis is a bacterial zoonotic disease of worldwide distribution. Little information is available regarding the occurrence of the disease in the Nile Delta provinces, Egypt. The present study investigated the seroprevalence of leptospirosis among cattle from Dakahlia province, Northern Egypt, and identified the individual variables factors associated with infection. To this end, a total of 600 serum samples from cattle of small stakeholders with various clinical manifestations possibly associated with leptospirosis were collected from different localities across Dakahlia province, Egypt. Sera were examined serologically via ELISA to investigate the occurrence of the disease among animals. Chi-square test and multivariable logistic regression analyses were applied to determine the association between hypothesized risk factors and the disease. Interestingly, our findings showed that 39.33% of the examined sera were positive for *Leptospira* antibodies, with significant differences among different localities. In addition, statistical analysis showed significant differences among age groups. Notably, the highest prevalence rate (22%) was observed in those aged between 3 and 5 years (*p* < 0.0001), whereas the lowest prevalence (2.66%) was reported in cattle <1 year old (*p* < 0.0001). Moreover, females had a significantly higher prevalence rate (35.33%) than males (4%) (*p* < 0.0001). Furthermore, our results showed significant differences in the occurrence of infection and reported clinical signs (*p* < 0.0001). Multivariable logistic regression identified repeated breeder and drop milk yield as the best predictors for prediction of ELISA results and linear discriminant analysis (LDA) model showed that overall classification accuracy of ELISA result using clinical signs and demographic data as predictors was 70.7%. The current study concluded a relative high prevalence of leptospirosis among cows bred in movable herds and households in the studied area and that age, repeated breeder and drop milk yield can be considered major risk factors associated with infection.

## 1. Introduction

Leptospirosis is a re-emerging zoonosis of worldwide distribution with increased prevalence in tropical, subtropical, and temperate countries [1]. This disease is caused by spirochetal pathogens of the genus *Leptospira*, which comprise pathogenic and saprophytic species. Although rodents are considered the main reservoirs [2,3], the epidemiological profile of the pathogen also includes a wide range of domestic and wild animals, besides humans, that can be considered natural reservoirs and carriers of *Leptospira*. As such, the pathogen is transmitted mainly by direct contact of mucosa or damaged skin to exposed water or soil contaminated with the urine of infected animals, subsequently colonizing the proximal renal tubules of various mammals [4]. According to genetic relatedness, numerous immunologically distinct serovars of *Leptospira* spp. have been identified and classified into over 300 serovars grouped into almost 30 serogroups [5]. Certain serovars may adapt to specific animal hosts, such as *L. interrogans* serogroup Hardjo serovar Hardjo in cattle, which appeared as an asymptomatic carrier animal. In contrast, the same animal species can become an accidental host of another serovar, such as *L. interrogans serogroup Pomona* serovar *Pomona, L. interrogans* serogroup *Grippotyphos* serovar *Grippotyphos* and *L. interrogans* serogroup *Icterohaemorrhagiae* serovar *Icterohaemorrhagiae*, as in the case of bovine leptospirosis [6,7].

Regarding its clinical impact, leptospirosis is definitely a cause of significant wealth and health losses in animals and humans [8]. In animals, the disease can range from insignificant and asymptomatic to deadly, depending on the causative species [9]. Among others, bovine leptospirosis has been associated with multiple symptoms that include septicemia, abortion, infertility, hemoglobinuria, drop in milk production, and mastitis [10,11]. Given its zoonotic potential, studies have shown that human leptospirosis presents with various signs, some of which are mild (i.e., fever, headache, and myalgia) and some that are severe (i.e., liver and kidney failure). In addition, death in some cases of human leptospirosis frequently occurs due to pulmonary hemorrhage [12,13]. Pathogen detection in a host has been considered one of the main approaches for controlling the infection [14,15,16,17,18]. Isolation of the causative agent can be used to definitively diagnose leptospirosis. However, bacterial isolation has been associated with some drawbacks considering that this method is time consuming and necessitates pathogen livability and viability of bacteria growth on culture media. As such, several serological techniques have been adopted for detecting anti-*Leptospira*l antibodies worldwide, which have provided several advantages over classical detection and isolation methods [19]. Among others, the enzyme-linked immunosorbent assay (ELISA) technique has been widely accepted as a standard serological test for detecting antibodies against a wide range of bacterial pathogens, including *Leptospira* spp. [20]. Considering this, ELISA has some advantages over other remaining techniques, such as the microscopic agglutination test (MAT). In this respect, ELISA is relatively sensitive, specific, and semi-automated, utilizes killed antigens, and allows for objective interpretation of results [21]. Moreover, ELISA has been considered practical, fast, and affordable and has a greater throughput. It is therefore not surprising that ELISA has been regularly used for initial serological screening in large-scale surveys for the identification of specific antibodies against *Leptospira* spp. [20]. Cattle play an essential social and economic role and are the primary source of meat and milk in Egypt [22]; however, the public health and economic consequences of leptospirosis have been a growing concern due to its clinical impact and implication in several reproductive disorders, including abortion, stillbirth, and birth of weak calves, aside from its role in reducing milk production [23]. After reviewing the available literature, we found that most of the previous studies in Egypt focused on leptospirosis in humans who had come in contact with animals [24]. Hence, recent investigations have been launched to detect *Leptospira*l antibodies in individuals with unexplained acute febrile illness and hepatitis using ELISA. These studies have demonstrated the need for determining the epidemiologic status of leptospirosis in Egypt, as well as identifying challenges in illness diagnosis [24]. However, few data have been available on the incidence and prevalence of leptospirosis in cattle across the major provinces contributing to cattle production in Egypt, particularly in the Nile Delta province, including Dakahlia Governorate [25,26]. Notably, the presence of some peculiarities in this area favors the existence and persistence of *Leptospira* in hosts and the environment (i.e., the presence of rodents, the presence of domestic animals raised in semi-extensive or extensive farms, the abundance of wetlands, such as ponds and irrigation canals, and the close contact between animals and humans during agricultural work, which may increase infection exposure and persistence and promote close contact with reservoirs, facilitating the maintenance of the epidemiological foci of the disease). Although the existence of these factors favor the emergence of the disease, epidemiological information regarding *Leptospira* spp. prevalence in Dakahlia remains limited, particularly among cattle from stakeholders. Given the aforementioned information, the current study primarily aimed to investigate the seroprevalence of bovine leptospirosis circulating within the Nile Delta province of Dakahlia via ELISA and explore the major epidemiological factors associated with the occurrence of the disease in this area.

## 2. Materials and Methods

### 2.1. Ethical Considerations

The study protocol was carefully reviewed and approved by the local guidance body on Research, Publication and Ethics of the Faculty of Veterinary Medicine, Mansoura University, Egypt, which complies with all the relevant Egyptian laws on research and publication. The institutional approval code of the study number is R/120 and it was approved on 20 December 2018.

### 2.2. Study Area, Sampling, Animal Data, and Clinical Examination

A cross sectional study to estimate the prevalence of leptospirosis among cattle showing one of the suspected clinical signs of leptospirosis was carried out. A cluster sampling was used as a sampling strategy with the individual animal as the primary sampling unit. The sample size was estimated as 267 animals using Win Epi 2.0 with expected prevalence of 50% and 6% accepted error and infinite population. This number of samples was multiplied by design effect of 2 [27] and so the sample size was estimated as 534 animals and this number was inflated to 600. The 600 samples were distributed among 4 districts of the governorate proportional to size. The total of 600 serum samples were collected from cattle of small stakeholders within Dakahlia province from January to December 2019. In accordance with their localities, 180, 144, 168, and 108 samples were collected from the Sherbin, Dikirnis, Belkas, and Mansoura areas, respectively. The choosing of the animals was carried out in a purposive way, focusing on households which had an animal with suspected leptospirosis. Regarding sampling, after informed consent was obtained from the animals’ owner, blood samples were collected by licensed veterinarians from cattle by puncturing the jugular vein with vacuum tubes without using an anticoagulant. Samples were then centrifuged at 10,000 rpm for 5 min to obtain the serum, which was stored in 1.5-mL microtubes at −20 °C until further testing.

### 2.3. Epidemiological Information and Clinical Signs

In this step, the demographic information of each animal, including the sampling date, age, sex, and locality, were recorded. The full details of the study cohort are shown in Table 1. Samples were collected from cattle that have shown at least one of the clinical signs suggesting leptospirosis, such as abortion, repeat breeder, bloody milk, and mastitis. The selection criteria of our study samples was in accordance with those described elsewhere [28,29]. Moreover, samples were collected from cattle with no history of previous vaccination against leptospirosis.

### 2.4. Serological Investigation Using ELISA

ELISA was performed on serum samples (*n* = 600) obtained from cattle using SERION ELISA classic *Leptospira* IgG (Order Nr.: ESR125G) lot SKI. BG manufactured by Institute Virion/Serion GmbH—D-97076 Wurzburg. This kit is used for the diagnosis of human leptospirosis but it was adapted for bovine leptospirosis using anti-bovine conjugate, (Prionics AG, Lelystad, The Netherlands).The principles and manufacturer’s procedures and adoption of these kits for bovine leptospirosis were followed using specific the previously mentioned anti-bovine conjugate as described elsewhere [30,31]. The procedure for manual ELISA testing was followed. Briefly, a total of 100 µL of diluted sample or controls were added to appropriate wells of microtiter test strips and then allowed to incubate in a wet chamber at 37 °C for 60 min. Wells were then washed three times and incubated using 300 µL washing solution, after which the incubation solution was removed by aspirating or shaking it out using a paper towel. With the exception of blank wells, the remaining wells were filled by 100 µL IgG conjugate (anti-bovine conjugate), then incubated in a wet chamber at 37 °C for 30 min followed by rinsing of all wells with washing solution after incubation. Thereafter, 100 µL of substrates (para-nitrophenyl-phosphate in solvent-free buffer) was added to each well, including the well for the substrate blank, after which the plates were incubated at 37 °C in a moist chamber for 30 min. Afterwards, 100 µL of sodium hydroxide was poured into each well as a stopping solution and the microtiter plate was gently shaken to mix. The results of the colored reaction were checked under an optical density of 405 nm against a blank substrate at 60 min using a Titertek Multiskan ELISA reader. The calculation the results for ELISA were performed as per the kits instructions (SERION ELISA classic *Leptospira* IgG/IgM).

### 2.5. Statistical Analyses

Data were organized, summarized, and then analyzed using SPSS version 18.0 (SPSS, Chicago, IL, USA). Results with *p* values < 0.05 were considered statistically significant. The Chi-square test (χ^2^) was used to compare frequencies of ELISA results (exposed or not exposed) in different age, locality, sex, and clinical signs. In addition, the association of ELISA result with the possible risk factors and clinical signs were assessed using binary logistic regression. Parameter estimates, including odds ratio and 95% confidence interval, were presented.

Regarding the potential individual variable risk factors detection, the binary logistic regression analysis was performed to elucidate the most distinctive predictors for ELISA results by using risk factors (age, locality, and sex) and clinical signs. The univariate binary logistics regression analysis was carried out firstly to determine significant risk factors (age group, sex and locality) which can be used as predictors for ELISA result (exposed or not exposed) and this showed that only age group was statistically significant at *p*-value < 0.05. Clearly, multivariate binary logistic regression analysis was not performed because only age was the significant predictor. In accordance with factors associated with clinical signs, the univariate binary logistics regression analysis was carried out to determine significant clinical signs (fever, repeat breeder, clinical mastitis, subclinical mastitis, bloody milk, abortion and drop in milk yield) that can be used as predictors for ELISA result (exposed or not exposed) and this showed that only repeat breeder and drop in milk yield were statistically significant at *p*-value < 0.05. The multivariate binary logistic regression analysis was then performed using the significant predictors from univariate binary logistics regression analysis, which included repeat breeder and drop in milk yield using the following equation:

Log(*p*/1 − *p*) = b_0_ + b_1_ × X_1_ + b_2_ × X_2_ + b_3_ × X_3_

In(odds) = odds of beef breeds & b = constant

Finally, the linear discriminant analysis (LDA) model was used to classify ELISA results (exposed or non-exposed) as dependent variables using age, locality, sex, and clinical signs as predictors (independent variables). The discriminant statistical function used for this analysis was as follows:

DF = V_1_ × 1 + V_2_ × 2 + V_3_ × 3 + … + V_I_ × X_I_

where: DF = discriminate function (score) of grouping of grouping variables, V = the standardized discriminant coefficient or loadings for the clinical signs (predictors), X = respondent’s score for the clinical signs, and I = the number of predictor variables. The discriminant function coefficient V or standardized form beta indicate the partial contribution of each clinical sign to the discrimination process.

## 3. Results

### 3.1. Clinical Manifestations

The present study showed that the exposed animals exhibited various clinical signs. Notably, among the 600 examined animals, 104 showed fever, 228 suffered an abortion, and 36 were found to be repeat breeders. Meanwhile, bloody milk, drop in milk yield, clinical mastitis, and subclinical mastitis were recorded in 32, 44, 72, and 84 animals, respectively.

### 3.2. Serological and Epidemiological Findings

This study found that 236 animals had antibodies against *Leptospira*, reflecting a seroprevalence of 39.33%. As shown in Table 2, cattle of small stakeholders from Dikirnis and Belkas presented the highest prevalence rate (11.33%), followed by those from Mansoura (8.67%), with animals from Sherbin locality showing the lowest recorded seroprevalence (8%).

The epidemiological findings and potential risk factors associated with occurrence of the disease are shown in Table 2, Table 3, Table 4, Table 5 and Table 6. As mentioned earlier, Chi-square analysis of different assumed determinants of animals had antibodies against *Leptospira* showed that age, sex, and clinical signs were significantly associated with the seropositivity to *Leptospira*. Regarding age, a significant association was observed between the frequency of ELISA-positive samples and age (*p* < 0.0001). As illustrated in Table 3, all cattle aged over 1 year old showed higher prevalence rates than those <1 year old. However, the highest prevalence rate (22%, 132/600) was observed among those aged 3–5 years, whereas the lowest prevalence rate was recorded among those cattle <1 year old (2.66%). Regarding sex as a potential variable among the studied animals (Table 4), a significant difference was observed between male and female cattle (*p* ≤ 0.0001). Notably, the highest infection rate was observed among female animals (35.33%), while male animals showed an infection rate of 4% (24/600). Moreover, a significant difference (*p* < 0.0001) was noted between the frequencies of positive results via ELISA and different clinical signs recorded (Table 5). In this concern, the highest frequency rate of leptospirosis was reported in cattle who exhibited bloody milk 75% (24/32) followed by those that experience a drop in milk production 72.72% (32/44) and repeat breeders 66.67% (24/36). However, as shown in Table 5, a low frequency rate 26.92% (28/104) has been recorded for cattle with fever. Moreover, the frequency rates of leptospirosis in clinical and subclinical mastitis cases were 38.89% (28/72) and 28.57% (24/84), respectively. The present study used Binary Logistic Regression to determine predictors for the ELISA test result. Using univariate analysis of risk factors, it was found that age was the significant predictor for this, while the results of multivariate binary logistic function for clinical signs related to the infection revealed that repeated breeder and drop milk yield were best predictors with estimates (1.9 and 1.7), respectively, and they seem to be distinct predictors that can be used to predict ELISA results (Table 6). Furthermore, as depicted in Table 7, LDA was conducted to classify ELISA results using clinical signs and demographic data as predictors. Accordingly, our findings showed that 304/364 (83.5%) negative cases were correctly classified, whereas 120/236 (50.8%) positive cases were correctly classified, with an overall classification accuracy of 70.7%.

## 4. Discussion

Cattle have an essential role from both social and economic aspects, and are the primary source of meat and milk [22]; however, the public health and economic consequences of leptospirosis are of growing concern due to the clinical impacts and its implication in several reproductive disorders, besides its role in reduction of milk production. Leptospirosis has been considered an alarming and re-emerging zoonosis with a worldwide distribution [32]. The disease is usually misdiagnosed as other tropical febrile diseases due to similarities in clinical manifestations. Although treatment regimens could be started based on clinical judgments, early diagnosis has become a vital guide for chemotherapeutic interventions [33]. As mentioned earlier, ELISA is an immunoassay based on the specific interaction of antibodies with their corresponding antigen. This immunoassay has been considered a very sensitive serologic test particularly suited for determining antibodies against a wide range of bacterial, viral, and parasitic illnesses [34,35,36]. The test strips of the SERION ELISA classic microtiter plate are coated with specific antigens of the pathogen of interest was used in the present work. The present study provides interesting findings in relation to the occurrence of leptospirosis in cattle from the Nile Delta province of Dakahlia, as well as baseline epidemiological information regarding the disease in the same area.

In the present work, the seroprevalence of leptospirosis was 39.33% (236/600). A review of the available literature at national level revealed that very limited studies have explored the status of leptospirosis from the same province [25,26]. Contrary to the present results, lower prevalence rates of bovine leptospirosis (*L. interrogans* serogroup Hardjo serovar Hardjo) were previously reported among cattle in other countries using ELISA, including 5.77% India [37], 3.5% and 8.44% in Nigeria 2011 [38,39] and 23.5% in Kenya between September 2016 and July 2017 [40]. In addition, other previous research in Pakistan reported a prevalence rate of 23.12% for bovine leptospirosis in cattle and buffalo using ELISA [8]. On the other hand, the seroprevalence obtained herein was similar to that reported in a previous study in Egypt, which found a seroprevalence rate of 37.6% (235/625) for *Leptospira* serovars among cattle from several governorates in Egypt using MAT [24]. However, other previous research in Egypt recorded slightly higher seroprevalence rates (44%) of leptospirosis in nine cattle from Mahalla City, Gharbeya Governorate, Egypt using MAT [9]. The same previous study [9] explored the cross-species surveillance of *Leptospira* in animals to discover the most common serovars in the studied area region, reflecting that wild and domestic mammals are key sources of pathogenic *Leptospira*. As mentioned previously, very limited studies have explored the status of leptospirosis from the same province [25,26]. In stark contrast to our present findings, a previous study [25] that examined a total of 97 urine samples from suspected cattle revealed a prevalence rate of 28.9% (28/97) and 45.4% (44/97) for leptospirosis among examined cattle using Dark Field Microscopy and staining via Silver Impregnation Methods, respectively. Another previous study [26] revealed a lower prevalence rate of 4.8% for *Leptospira interrogans* serogroup Icterohemorhagia serovar Icterohemorhagia among a total number of 353 serum samples from cattle in Dakahlia province using the complement fixation test. Likewise, another study in cows from Kafrelsheikh governorate, Egypt, reported very low seroprevalence (3.6%) of leptospirosis caused by *L. interrogans* serogroup Hardjo serovar Hardjo using ELISA [41]. Furthermore, a recent outbreak of leptospirosis in a total of 45 sheep had been reported in a previous study [42], which had been the first epidemic in northern Egypt. Contrary to the results of the current study, very high prevalence of bovine leptospirosis had been previously reported in other countries, including India (87.0%) [43] and Poland (89.9%) [44]. This difference in leptospirosis prevalence rates between our study and those mentioned earlier might have been attributed to multiple factors, including geographic location, climatic conditions, sample size, the performed serological test and its sensitivity, abundance of rodents, herd management, and animal density considering that the presence of a dense population of cattle infected with *Leptospira* spp. may contribute to environmental contamination and dissemination of infection [4,45,46]. It is therefore unsurprising to suggest that the transmission dynamics of the disease is exceedingly complex and variable and seems to be associated with environmental conditions [45,46].

Interestingly, the present study investigated multiple individual variable factors, including locality, age, sex, and reported clinical signs and their potential association with infection occurrence. Regarding locality, the prevalence rate of leptospirosis was significantly higher (11.33%) in cattle of stakeholders from Dikirnis and Belkas districts compared to those from Mansoura (8.67%) and Sherbin districts (8%). The present findings are consistent with several previous studies, which hypothesized that leptospirosis was highly associated with exposure to rice fields and contaminated surface water [41,47]. However, this difference among studied localities might have been due to variations in agricultural practices and the fact that animals from Dikirnis and Belkas districts usually drink from surface water channels used for land irrigation. In addition, several rice fields are present in the governorate, whereas animals from Mansoura and Sherbin used tape water (treated) for drinking.

Regarding the four age groups studied, Table 3 revealed that the prevalence rate was significantly higher in cattle aged 3–5 years (22%, 132/600), followed by those aged 1–3 years (8.67%, 52/600), with those below 1 year old showing a lower prevalence rate (2.66%, 16/600). This result is consistent with several previous studies [4,48,49] that found higher prevalence rates among cattle in the 3- to 5-year age group compared to other age groups. Previous research revealed that leptospirosis seroprevalence, using ELISA, in cattle gradually increased with the age of the animals [39]. A possible explanation could be the persistence of antibodies in animals over a long duration and the longer exposure period, which increase the probability of animals becoming infected and consequently playing a major role as chronic carriers and shedders of *Leptospira* spp. into the environment [38]. As depicted in Table 4, our results revealed that female cows (35.33%) had a significantly higher prevalence rate compared to male cows (4%). These findings are supported by a previous study [8], which revealed that females were more prone to contracting the infection than males using ELISA. In contrast, much previous research has reported that males had higher prevalence rates of leptospirosis than females, although the difference were not significant [41,48,49]. The possible explanation for these contraindicating findings and reported difference in relation to sex remains unknown [48]. However, the fact that most of the samples examined in the current study were collected from female cows cannot be neglected given its potential influence on our findings.

Regarding clinical signs as potential individual variable factors, Table 5 showed that the prevalence rate of leptospirosis was significantly higher (75%) in cattle that suffered from bloody milk, followed by those that showed signs of a drop milk yield (72%). Meanwhile, the prevalence rate of leptospirosis in animals with mastitis cases was 33.3% (52/156). In addition, the prevalence rate of leptospirosis in animals with reproductive disorders was 66.67% and 33.33% in repeat breeders and cattle that suffered an abortion, respectively. Our results are consistent with those reported in several previous studies [50,51], which showed that leptospirosis in cattle may be associated with acute, subacute, or chronic mastitis. In fact, a peculiar type of mastitis is another distinctive form of leptospirosis where the whole udder is affected and there is abnormal milk in all four quarters. Milk drop syndrome occurs in lactating cows given that the proliferation of the organism is restricted to the lactating mammary gland. These findings are consistent with previous reports, which concluded that *Leptospira* spp. have a chronic presentation in bovines and might result in severe reproductive problems, including abortion, stillbirth, and low fertility [4,52]. In fact, all the examined cattle in this study were not vaccinated, which is consistent with the hypothesis that vaccination plays an important role in the control of leptospirosis and may significantly reduce the occurrence of clinical symptoms in the herd, including reproductive orders [53,54]. Collectively, multivariable logistic regression showed identified repeated breeder and drop milk yield as the best predictors for prediction of ELISA results. Meanwhile, a previous study [8] identified sex, fodder type, jaundice status, and socio-economic status of the animal’s owner as key risk factors for leptospirosis seroprevalence dynamics (OR > 1).

## 5. Conclusions

The present study concluded that the seroprevalence of bovine leptospirosis in the Delta region was relatively high. Our study uncovered some interesting baseline epidemiological information that could be associated with the occurrence of the disease in the studied animals. Among others, age, repeated breeder and drop milk yield had been identified as key potential individual risk factors for leptospirosis. With the considerable prevalence of bovine leptospirosis and the assumed determinants appearing as potential risk factors, infected animals might act as reservoirs for cross-species transmission of the infection. Further large-scale investigations aiming to explore leptospirosis in cattle, either symptomatic or asymptomatic, and other animal reservoirs in the Egyptian are warranted for combating such zoonosis.

## Figures and Tables

**Table 1:** Locality and ages of examined animals.

Locality	Total	Sex		Age			
		Male	Female	Up to One Year	1–2 Year	3–5 Year	>5 Year
Dikrins	144	24	120	12	20	104	8
Belkas	168	28	140	20	36	100	12
Mansoura	108	20	88	8	32	48	20
Sherbin	180	24	156	24	72	64	20
Total	600	96	504	64	160	316	60

**Table 2:** Prevalence of leptospirosis in cattle according to locality.

Locality	No. of Examined	ELISA Positive	ELISA Negative	χ^2^	*p*-Value	Prevalence (%)
Sherbin	180	48	132	76.54	<0.0001 **	8%
Dikrnis	144	68	76	0.680	0.409 N.S	11.33%
Belkas	168	68	100	11.44	0.0007 **	11.33%
Mansoura	108	52	56	0.167	0.683 N.S	8.67%
Total	600	236	364	35.76.81	<0.0001 **	39.33%

**: Highly significant. N.S: Non-significant.

**Table 3:** Prevalence of leptospirosis in cattle according to age group.

Age Group	No. of Examined	ELISA Positive	ELISA Negative	χ^2^	*p*-Value	Prevalence (%)
<1 year	64	16	48	30.03	<0.0001 **	2.66%
1–3 year	160	52	108	37.81	<0.0001 **	8.67%
3–5 year	316	132	184	16.46	<0.0001 **	22%
>5 year	60	36	24	4.03	0.044 *	6%
Total	600	236	364	35.76.81	<0.0001 **	39.33%

**: Highly significant. *: Significant.

**Table 4:** Prevalence of leptospirosis in cattle according to sex.

	No. of Examined	ELISA Positive	ELISA Negative	χ^2^	*p*-Value	Prevalence (%)
Male	96	24	72	46.02	<0.0001 **	4%
Female	504	212	292	24.76	<0.0001 **	35.33%
Total	600	236	364	35.76.81	<0.0001 **	39.33%

**: Highly significant.

**Table 5:** Frequency of seroprevalence of leptospirosis among cattle according to clinical signs.

Clinical Signs	NO. of Animals Examined	ELISA Positive	ELISA Negative	χ^2^	*p*-Value	Frequency (%)
Fever	104	28	76	42.48	<0.0001 **	26.92‰
Abortion	228	76	152	49.34	<0.0001 **	33.33%
Repeated Breeder	36	24	12	6.72	0.009 **	66.67%
Bloody milk	32	24	8	14.06	0.0002 **	75%
Subclinical Mastitis	84	24	60	29.16	<0.0001 **	28.57%
Clinical Mastitis	72	28	44	6.25	0.012 *	38.89%
Drop milk yield	44	32	12	16.41	0.0001 **	72.72%
Total	600	236	364	35.76.81	<0.0001 **	39.3%

**: Highly significant. *: Significant.

**Table 6:** Multivariate analysis of risk factors associated to *Leptospira* spp. infection in cattle.

Predictors	B	S.E.	Wald	D.F	Significance	Odd Ratio	95.0% C.I for EXP(B)	
							Lower	Upper
Repeat breeder	1.923	0.823	5.460	1	0.019	6.841	1.363	34.324
Repeat breeder (yes)	−1.923	0.823	5.460	1	0.019	0.146	0.029	0.733
Drop milk yield	1.651	0.702	5.532	1	0.019	5.212	1.317	20.629
Drop milk yield (yes)	−1.651	0.702	5.532	1	0.019	0.192	0.048	0.759
Constant	−0.670	0.185	13.073	1	0.000	0.512		

**Table 7:** Results of animal classification into infected and non-infected.

ELISA		Predicted Group Membership		Total
		Infected	Non-Infected	
Count	Infected	120 (50.85%)	116 (49.15%)	236 (100%)
	Non- infected	60 (16.48%)	304 (83.52%)	364 (100%)

## Data Availability

The data that support the findings of this study are available on request from the corresponding author.
